# The Impact of the COVID-19 Pandemic on China's Airline Industry

**DOI:** 10.3389/fpubh.2022.865463

**Published:** 2022-05-27

**Authors:** Yuan Zhang, LinChuang Zhu, Feng Hao

**Affiliations:** School of Economics, Tianjin University of Commerce, Tianjin, China

**Keywords:** China, airline industry, COVID-19, full-service airline, low-cost airline, stock prices

## Abstract

**Background:**

The COVID-19 pandemic has posed a great challenge to the development of China's airline industry. Although the existing literature has analyzed the economic impact of the pandemic on the airline industry from different perspectives, it remains to be further studied given the operating characteristics of different types of airlines in China.

**Methods:**

Using a new perspective of heterogeneous airline service models, this study collects high-frequency data on stock prices on six sample airline companies (including both full-service airlines and low-cost airlines) in China over 519 trading days, from August 1, 2019 to September 15, 2021, and identifies structural change points for each company's stock price using the Quandt-Andrews test. The outcome is used to construct an econometric model to quantify the economic impact of the pandemic on different airlines' stock prices under different structural changes.

**Results:**

All results have passed the Quandt-Andrews test. The impact coefficient of full-service airlines is negative, while that of low-cost airlines is positive. Most of them have passed the test at the significance level of 10%.

**Conclusions:**

All Chinese airlines experienced significant sudden changes in stock prices due to the pandemic, but there are sectoral differences in the order of the sudden changes, with full-service airlines experiencing structural changes much earlier than low-cost airlines. In addition, the impact of the pandemic on stock prices varies across airline types, with a negative impact on full-service airlines and a significant positive effect on most low-cost airlines.

## Introduction

In 2020, the COVID-19 pandemic swept the whole world and exerted a significant impact on the global society and major industries. As of September 2021, the COVID-19 pandemic has affected over 200 countries in the world with more than 23 million confirmed cases accumulated, causing more than 470 million death (1). Airline industry is vulnerable to major external events such as energy crisis and major public health. This has had a huge impact on the global civil aviation industry. In 2020, at the beginning of the pandemic, China's airline industry experienced a cliff-like decline. The entire industry completed a passenger traffic of 417,778,200, a decrease of 36.7% compared with the year 2019 (2). The pandemic caused a decline in passenger flow, the number of flights, and the number of routes offered, which caused unprecedented shock to the whole industry. The Airline industry is very sensitive to public health emergencies. Since the beginning of the twenty first century, major global public health events have erupted from time to time, such as severe acute respiratory syndrome (SARS) in 2003 and the influenza A virus (H1N1) in 2009. The impact of public health emergencies on China's airline industry has been turned out to be greater than on the country's railway system and highway system. Facing the uncertain risks brought by the COVID-19 pandemic, it is necessary to use effective means to assess and resolve them, which will help the recovery and sustainable development of aviation industry and related industries.

Due to the different outbreak timings of different pandemics, the economic impact of the COVID-19 pandemic on airlines may vary with time. At the beginning of 2020, COVID-19 spread in China. After a series of efforts by the Chinese government, the pandemic has been effectively controlled in mid-2020. The Chinese government has successively issued the exemption of civil aviation development funds paid by civil aviation companies (3), taxpayers are exempted from VAT on income derived from transportation of key protection materials for pandemic prevention and control (4). The longest carry-forward period for losses incurred by enterprises in difficult industries that are more affected by the pandemic in 2020 will be extended from 5 to 8 years (5). Airport management agencies are exempt from aeronautical business charges and ground service charges for the implementation of major transportation missions, and air traffic control units are exempt from approach command fees and route fees; reduce domestic, Hong Kong, Macao and Taiwan regions, and foreign airline airports and air services.

In addition to time uncertainty, the impact of public health emergencies on the airline industry and its subsequent survival are also related to its business model (6). Existing airlines are mainly divided into two categories: full-service airlines and economy airlines. Economy airlines are also known as low-cost airlines (Low-Cost Carriers). Low-cost airlines generally use a single-type fleet. The model can save the proportion of the number of aircrews and mechanics required by airlines. On the one hand, it improves the efficiency of daily operation and maintenance of the aircraft, and on the other hand, it saves human resources. Secondly, compared with the full-service type of airlines, low-cost airlines generally have no frills, that is, they generally do not provide passengers with in-flight catering and entertainment services or only provide basic beverages or snacks, thus eliminating the need for in-flight food heating equipment to make the aircraft cabin layout the space that becomes simple and free can increase the number of seats, and most importantly, it saves the company food procurement and labor costs. Therefore, low-cost airlines generally rely on cost advantages to implement long-term low-cost strategies on various routes and formulate looser ticket usage conditions. Due to the significant reduction in operating costs, the fares of low-cost airlines are generally lower than that of full-service airlines. The second category of civil aviation is full-service airlines. Full-service airlines generally have more than one type of aircraft. According to different sales and travel plans, there are different aircraft fleets for adjustment, and full-service airlines generally to provide differentiated services, in addition to some basic services including a certain limited baggage allowance, it will provide passengers with on-board catering and entertainment services, which greatly improves the comfort and happiness of customers. Full-service airlines tend to adopt hub-and-spoke route networks, establish the status of airport hubs, and use density economies and scope economies to form barriers to entry for other carriers that provide homogeneous products, including take-off and landing times, use of airport facilities, etc. All aspects have advantages. Generally, a wide and balanced domestic and international route network has been formed during the operation of full-service airlines for many years, and a balanced and complementary route network has been formed. Compared with economical airlines, they have absolute route advantages.

### Literature Review

The impact of the COVID-19 pandemic on the airline industry has been discussed from the perspectives of national policies, aviation markets, and financial markets. In terms of national policy, Chinazzi et al. (7) use a global meta-population disease transmission model to predict the impact of travel restrictions on the domestic and international spread of COVID-19. The results of the study indicate that travel bans are significantly positive in reducing imported cases. However, it has only a slight impact on the trajectory of the pandemic. Combining measures such as community management and control will help curb the further spread of the pandemic. Iacus et al. (8) also study the impact of the travel ban on the aviation industry and further evaluated the socio-economic benefits of this impact. The authors find that the impact of the travel ban on the aviation industry may cause global GDP losses in 2020 up to 1.41–1.67%, and may result in unemployment of 2,500–3,000 million workers.

As for the impact of the COVID-19 on China's airline industry, Li et al. (9) use a gradient boosting decision tree to study the dynamic impact of COVID-19 on China's intercity tourism and find that during the pandemic, China's intercity travel was reduced by nearly 50%. The analysis results of air transportation capacity, traffic flow, revenue, and international market show that the impact of COVID-19 on airlines is different. The less-funded airlines are negatively affected (9); while airlines that focus on the domestic market and economy class and receive more funding have limited impact (10). The impact of the COVID-19 pandemic on cargo airlines and passenger airlines is somewhat different. The reason is that pandemic prevention measures are mainly for air passengers, while air cargo is less affected (11). The risk index is used to quantify the risk of imported cases on inbound international flights. The results show that after China implements strict control on inbound flights, the number of imported cases has dropped by about 50%, while Hong Kong, Taiwan and other areas with dense international flights have a higher risk of imported cases during the COVID-19 pandemic, the impact of the three modes of transportation including high-speed rail, air and long-distance buses on the spread of the pandemic. It is found that the speed of the pandemic's spread was significantly related to the number of cities' airports and high-speed rail stations, but its correlation with the total number of confirmed cases is very high (12).

In addition, the COVID-19 pandemic also has a certain impact on the economic and financial markets of the aviation industry. The COVID-19 pandemic has greatly reduced travel demand, affected investor expectations, and caused a negative impact on airline stock prices (13). In terms of the impact of global air transport of different scales, the impact of COVID-19 on international flights is much higher than domestic flights (14). Santos et al. (15) use a two-step regression method to study the differential impact of COVID-19 on air travel demand. The study finds that short-density and low-density routes are one of the most affected aviation markets, while commercial routes are more affected than leisure routes.

Uncertainty is the main driving factor of many economic recessions, and the economic shocks related to uncertainty spread over time (16). These uncertain shocks may be caused by many factors, including financial crisis, terrorism-related events, disease outbreaks and natural disasters. Empirical economics research has already discussed the theoretical problems of uncertain shocks on macroeconomic conditions (17), and the increase of uncertainty often reduces the actual activities in the economy (18). The stock market is a barometer of economic development. The change of stock price can not only reflect the economic performance, but also predict the future economic development situation to a certain extent. The stock price may have structural change under the impact of uncertainty, and produce differences among different companies within the industry. Most studies often use the method of event analysis to study specific time points and build econometric models to quantify economic impact (19, 20). This paper will use this method.

Although some studies have discussed the impact of the COVID-19 pandemic on airlines, the heterogeneity of different types of airlines has not been studied in depth. In addition, the economic impact of different countries and different types of airlines of the COVID-19 pandemic may be different. Therefore, this article uses a new perspective of airline service models to study the heterogeneous impact of the COVID-19 pandemic on airlines with different service modes, selecting China daily average price of representative airlines quantifies the impact of the COVID-19 pandemic on the airline's economic impact.

For different time and business models, taking the opportunity of analyzing the impact of the pandemic on the aviation industry, fully understanding and judging the impact of major external events on the civil aviation industry, systematically planning effective response measures, being good at turning crises into opportunities, and reducing the negative impact of external events on the civil aviation industry. It is of great significance for reducing the volatility of China's civil aviation development, improving the endogenous resilience of the civil aviation industry in response to external shocks, and then becoming a powerful civil aviation country. At present, there is no relevant research using historical industry data and econometric models for empirical analysis. This research will make an exploratory attempt for the first time.

## Materials and Methods

### Data Description

In order to effectively identify the timing of structural changes in the full-service and economic aviation industries under the impact of the pandemic, and to compare the changes in structural changes of different types of aviation industries, this paper selects representative companies in China's full-service and economic aviation industries from 2019 to 2021 and conduct empirical analysis of the time series of average daily stock prices.

For the analysis of structural change in the stock prices of Airline companies in China, we select our sample period as from Aug. 1, 2019 through Sept. 15, 2021 covering 519 trading days and we consider average daily stock prices for 6 airline companies. The full-service airlines include China Southern Airlines Group Limited (China Southern Airlines) (hereinafter referred to as China Southern Airlines), China Eastern Airlines (hereinafter referred to as Eastern Airlines) and China International Airlines Co., Ltd. (AIR CHINA) (hereinafter referred to as Air China). The economy type (lower Cost-based) airlines include Spring Airlines (hereinafter referred to as Spring Airlines), Huaxia Express Airlines (hereinafter referred to as Huaxia), and Shanghai Juneyao Airlines (hereinafter referred to as Auspicious), the data are all from the “Rice Quant.”

### Analytical Strategy

**Figure 1:**
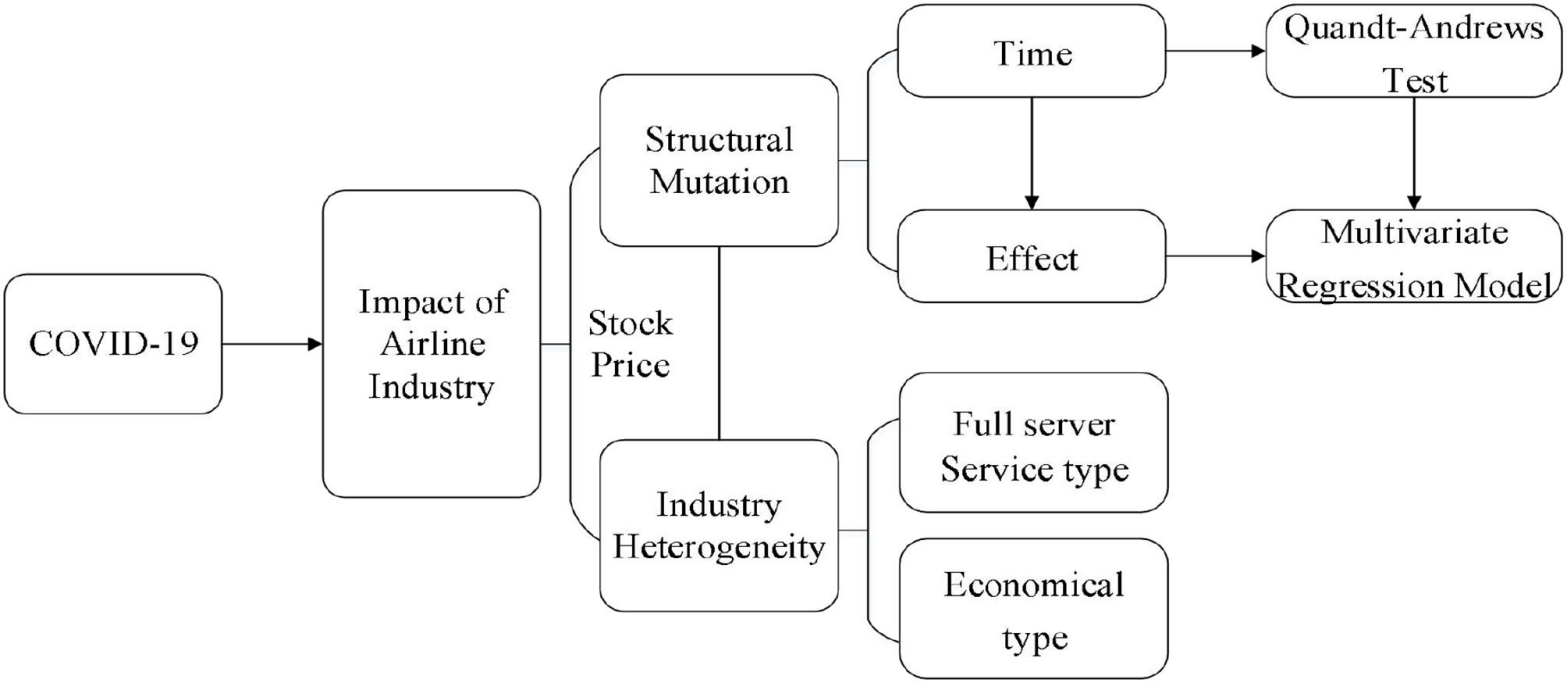
Research Framework.

First, the Quandt-Andrews test is used to identify structural change points of each company's stock price during the pandemic, which overcomes the inability of the traditional Chow test to identify unknown change points. Furthermore, a quantitative impact model was constructed to examine the economic effects of the pandemic on the stock prices of various companies under the impact of different structural change points. Finally, according to the regression analysis of the impact of the pandemic on the aviation industry, it compares the impact of different types of airlines in different countries, and provides relevant conclusions and policy recommendations, in order to further supplement and expand existing research. The research framework is shown in Figure 1.

### Quandt-Andrews Test

Structural change is generally defined as a sudden or sharp change in economic activity from one state to another from a certain point (year) due to major changes in economic conditions, such as policy transformation, natural disasters, oil crisis, etc. change often changes the parameters of economic variables (21). Because different types of airlines have different business models and economic strengths, the impact of the COVID-19 pandemic may have different impacts on their stock prices. In order to accurately identify the time of the sudden change of airline stock prices under the impact of the COVID-19 pandemic, this paper further conducts a parameter stability test analysis on the time series data of the daily average prices of two types of representative airline stocks. The parameter stability test is also called the variable structure test, which aims to test the statistical significance of the change of model structure parameters before and after a single or multiple time points. By comparing the statistical difference before and after the time point, it is inferred whether there is a structural change at that time point. In the field of economics and finance, the parameter stability test method represented by the Chow test has been widely used (22). This method has a good effect in testing the sudden change characteristics of economic indicators at a given time. However, because the test requires that the tested model does not miss important variables, and there is no systematic error in the model form setting, it also requires that the explanatory variable is not correlated with the random disturbance term, and each sub-interval must be satisfied during the test. At least as many samples as the estimated parameter, otherwise it may cause the estimation result to be biased, so the Chow test has greater limitations in its application.

In real economic issues, the timing of structural changes of most economic indicators is often unknown. This requires more flexible judgment and monitoring through other parameter stability testing methods. Aiming at the shortcomings of Chow test, Quandt and Andrews proposed a test method for unknown change points. This test method overcomes the inherent defect of Chow test requiring known change points. It can test the specified model data in one or more change points of unknown structure may exist on the value interval (τ _1_, τ _2_). In comparison, Chow test only test ([tau]_1_, [tau]_2_) whether a structural change occurs between two specified date or observations. According to Quandt-Andrews method, we divide the interval into *k* parts, make Chow test for each part, and then summarize it into a test statistic to check whether there is a structural change between τ _1_ and τ _2_.

Quandt-Andrews statistic test point change includes three types: ① Maximum statistic, i.e., by *Chow* obtained test *k* th *F* maximum statistics; ② *Exp* statistic, the test output result includes the *F* statistic value and *LR* statistic value of these three statistics; ③ *Ave* statistic, i.e., by Chow been tested in *k* th Simple arithmetic average of *F* statistic. Each Quandt-Andrews statistic can be divided into two statistics: Likelihood ratio *F* statistic (*Likelihood Ratio F-Statistic*) and *Wald-F* statistic (*Wald, the F-Statistic*). The likelihood ratio *F* statistic is a statistic based on the comparison of the residual sum of squares of the constrained and unconstrained models, while the *Wald-F* statistic is calculated according to the standard Wald test, which limits the coefficients of the model parameters. In the ordinary linear model, the two statistics are the same. A single Chow split point test statistic can be summarized into three different statistics: supremum statistic or maximum statistic (*Sup or Maximum Statistic*), *Exp* statistic and *Ave* statistic (23, 24).

The maximum statistic is a statistic obtained by taking the maximum value of a single statistic obtained in the test, namely:


(1) $$\begin{array}{r} \max F=\max_{\tau 1\geq \tau \geq \tau 2}F\left(\tau \right) \end{array}$$

*The* form of *Exp* statistics is as follows:


(2) $$ExpF=\ln(\frac{1}{k}\sum_{\tau =\tau_{1}}^{\tau_{2}}\exp\left(\frac{1}{2}F(\tau )\right))$$

*The Ave* statistic is a simple average of a single statistic obtained in the test:


(3) $$\begin{array}{r} AveF=\frac{1}{k}\overset{\tau_{2}}{\underset{\tau =\tau_{1}}{\sum }}F\left(\tau \right) \end{array}$$

It should be noted that the distributions of the above three types of statistics are non-standard. For this, Andrews (23) derives their true distributions. On this basis, Hansen (25) gives the approximate gradual changes of the three types of statistics. Near *p-* value, but the distribution of these statistics is generated between close to the sample starting point τ _1_ and close to the sample end point τ _2_, which makes the distribution of these statistics degenerate. To solve this problem, the usual approach is to the first and last 15% of the samples are eliminated.

### Econometric Model

Although the Quandt-Andrews test provides an effective technical tool for identifying change points of airlines under the impact of the pandemic, it cannot further quantify the economic effects of the impact of the COVID-19 pandemic on airlines. The occurrence of major emergencies is unpredictable. The COVID-19 pandemic will affect investor sentiment and change their trading decisions and behaviors, which will have a short-term impact on the stock market. In addition, the development of the pandemic has also restricted domestic and foreign travel, changed the expected itinerary of residents, which will have a certain impact on airline operations. In view of the dynamic nature of the autocorrelation and mutual influence of the pandemic, in order to accurately measure the economic impact of the COVID-19 pandemic on the stock prices of different types of airlines, this paper adopts the multivariate regression model (MVRM) with dummy variables (20, 26). This model not only provides a forward-looking measure of the uncertainty in the next period, but also fully considers the differential impact before and after public emergencies (27). The following measurement model is constructed:


(4) $$\begin{array}{r} R_{it}=\gamma_{i}+\alpha_{i}R_{it-1}+\beta_{i}\theta_{t}\left(i\right)+\epsilon_{it} \end{array}$$

Among them, represents the price of the *i-* th aviation stock at time *t*, θ_*t*_(*i*) represents the dummy variable that changes before and after the structural change point of the COVID-19 pandemic of the stock, β_(_*i*)represents the impact coefficient of the pandemic on the airline, and α_*i*_ represents the first-order autocorrelation coefficient of the *i* stock, γ_*i*_ is the intercept term, ε_*it*_ is a random error term and follows a normal distribution N(0, σ^2^).

### Robustness Test

In order to test the robustness of the estimation results, this paper adjusts the sample period, shortens the time window, and deletes 10% of the samples before and after the test.

## Results

### Impact on the Airline Industry Structural Change Points

Table 1 shows the descriptive statistical results of six airlines. There are great differences between the two types of airlines in terms of mean value and standard deviation. The share prices of full-service airlines have little difference, and their mean values are lower than those of economy airlines. The standard deviation of economy airlines is higher, and the stock price fluctuates more than that of full-service airlines.

**Table 1:** Descriptive statistical results.

**Industry type**	**Company name**	**Obs**	**Mean**	**Std. Dev**.	**Min**	**Max**
Full server (Service type)	China Southern Airlines	519	6.091	0.584	4.983	7.330
	Eastern Airlines	519	4.914	0.431	4.042	5.848
	Air China	519	7.741	0.787	6.326	9.845
Economical (Low cost type)	Spring and Autumn	519	47.221	9.268	29.995	68.204
	Huaxia	519	11.020	3.111	6.533	18.229
	Auspicious	519	12.455	2.162	8.870	17.403

In order to further investigate the correlation of stock prices among companies, the correlation coefficient matrix is given in Table 2. There is a strong positive correlation between full-service airlines, and the correlation coefficient is more than 0.9. In contrast, the correlation between economy airlines is slightly weak, and only auspicious has a large positive correlation with full-service airlines, with a correlation coefficient of more than 0.7.

**Table 2:** Correlation coefficient matrix.

**Company name**	**China Southern Airlines**	**Eastern Airlines**	**Air China**	**Spring and Autumn**	**Huaxia**	**Auspicious**
China Southern Airlines	1.000					
Eastern Airlines	0.947	1.000				
Air China	0.942	0.912	1.000			
Spring and Autumn	0.385	0.474	0.414	1.000		
Huaxia	−0.003	0.170	0.088	0.774	1.000	
Auspicious	0.736	0.777	0.771	0.627	0.228	1.000

Based on the time-series data, using Quandt-Andrews test for full-service and Chinese economy (low-cost type) airlines parameter obtain structural discontinuity stability test, calculation of formula (1)—of formula (3) statistic size and get the approximate asymptotic *p-*value, the test results are shown in Table 3.

**Table 3:** Quandt-Andrews inspection results of two types of Chinese airlines.

**Industry type**	**Company name**	**Change date**	**Statistics**		***P*-value**
Full server (Service type)	China Southern Airlines	2/03/2020	Maximum LR F-statistic	390.994	0.000
			Maximum Wald F-statistic	390.994	0.000
			Exp LR F-statistic	189.916	0.000
			Exp Wald F-statistic	189.916	0.000
			Ave LR F-statistic	73.883	0.000
			Ave Wald F-statistic	73.883	0.000
	Eastern Airlines	2/03/2020	Maximum LR F-statistic	184.587	0.000
			Maximum Wald F-statistic	184.587	0.000
			Exp LR F-statistic	87.172	0.000
			Exp Wald F-statistic	87.172	0.000
			Ave LR F-statistic	38.068	0.000
			Ave Wald F-statistic	38.068	0.000
	Air China	2/03/2020	Maximum LR F-statistic	168.196	0.000
			Maximum Wald F-statistic	168.196	0.000
			Exp LR F-statistic	78.980	0.000
			Exp Wald F-statistic	78.980	0.000
			Ave LR F-statistic	38.413	0.000
			Ave Wald F-statistic	38.413	0.000
Economy (Low-cost type)	Spring and Autumn	11/16/2020	Maximum LR F-statistic	1,559.046	0.000
			Maximum Wald F-statistic	1,559.046	0.000
			Exp LR F-statistic	774.274	0.000
			Exp Wald F-statistic	774.274	0.000
			Ave LR F-statistic	555.285	0.000
			Ave Wald F-statistic	555.285	0.000
	Huaxia	7/06/2020	Maximum LR F-statistic	1,386.968	0.000
			Maximum Wald F-statistic	1,386.968	0.000
			Exp LR F-statistic	688.594	0.000
			Exp Wald F-statistic	688.594	0.000
			Ave LR F-statistic	591.873	0.000
			Ave Wald F-statistic	591.873	0.000
	Auspicious	3/03/2021	Maximum LR F-statistic	373.966	0.000
			Maximum Wald F-statistic	373.966	0.000
			Exp LR F-statistic	182.525	0.000
			Exp Wald F-statistic	182.525	0.000
			Ave LR F-statistic	106.311	0.000
			Ave Wald F-statistic	106.311	0.000

Table 3 shows the Quandt-Andrews test results of 6 full-service and economical airlines in China. It can be seen that at a significance level of 1%, each airline's Quandt-Andrews statistic has passed the significance test, indicating that each airline has structural change points, and the numerical order has a common characteristic law, that is, the maximum statistic > *Exp* statistic > *Ave* statistic. On the order of structural change, there is a disparity within the airline industry, which is the structural change in full-service airlines occurs well before the economy (low cost type) airlines. For all full service airlines, structural breaks occurred on the same date, which is February 3, 2020. In contrast, there is a certain lag in the structural change date for economy (low-cost) airlines, more than one quarter later than that of full-service airlines. Structural change for Huaxia occurred as early as June 2020. For Auspicious and Spring, structural change occurred, respectively, in Nov. 2020 and May 2021. It can be seen that the impact of the COVID-19 pandemic on China's full-service aviation is the same, but the impact on the economy (low-cost) type is quite heterogeneous.

### Impact of the Pandemic on Airline Stock Prices

Table 4 shows that structural changes caused by the impact of the pandemic have a significant impact on most airline stock prices. The coefficients of full-service airlines are all negative. With the exception of Eastern Airlines, China Southern Airlines and Air China both passed the test at a significance level of 10%. The impact coefficients are −0.025 and −0.119, respectively, verifying the negative effects on full-service airlines in the early stage of the outbreak. From the comparison of the three companies, as Air China has the largest business scale, the impact of the pandemic on its negative impact is also the most prominent. In February 2020 during the Chinese New Year holiday, with the spread of COVID-19 pandemic, most full-service airlines, including Air China had to be canceled, including a large number of domestic and international flights, lower flight prices, air traffic presented cliff-style decline. The impact coefficient of China Eastern Airlines is −0.011 and is not significant. This is because China Eastern Airlines has adopted measures such as adjusting its operating strategy, actively seeking policy support, implementing strict cost control measures, cutting or delaying investment plans, etc., to ensure the company's cash flow is stable. And China Southern Airlines and Air China are also trying to minimize the impact of the pandemic through domestic and international linkages, passenger and cargo linkages, and resource protection linkages. However, due to the type of business, the negative impact it suffers is greater than that of China Eastern Airlines.

**Table 4:** The impact of the pandemic on the stocks of Chinese airlines.

**Industry type**	**Company name**	**Parameter**	**Coefficient**	**Std. Dev**.	**t statistic**	***P*-value**
Full server (Service type)	China Southern Airlines	α_*i*_	0.967	0.011	88.990	0.000
		β_*i*_	−0.025	0.015	−1.680	0.094
		γ_*i*_	0.220	0.075	2.950	0.003
	Eastern Airlines	α_*i*_	0.968	0.010	92.800	0.000
		β_*i*_	−0.011	0.011	−1.050	0.296
		γ_*i*_	0.162	0.056	2.880	0.004
	Air China	α_*i*_	0.798	0.027	30.150	0.000
		β_*i*_	−0.202	0.054	−3.740	0.000
		γ_*i*_	1.717	0.229	7.500	0.000
Economy (Low-cost type)	Spring and Autumn	α_*i*_	0.970	0.009	102.730	0.000
		β_*i*_	0.540	0.178	3.040	0.002
		γ_*i*_	1.237	0.387	3.190	0.001
	Huaxia	α_*i*_	0.984	0.009	116.290	0.000
		β_*i*_	0.067	0.053	1.260	0.209
		γ_*i*_	0.140	0.070	1.990	0.047
	Auspicious	α_*i*_	0.983	0.008	125.710	0.000
		β_*i*_	0.069	0.038	1.810	0.071
		γ_*i*_	0.195	0.092	2.120	0.034

In contrast, the impact of the pandemic on Chinese economy airlines has a certain time lag. Compared with full-service companies, the impact of the pandemic has not negatively affected the average daily stock price of its stocks, but has a certain positive effect. The impact of the COVID-19 pandemic on the three companies of Spring, HuaXia, and Juneyao Airlines are, respectively, 0.540, 0.067, and 0.069, of which Spring Airlines has the strongest impact. The early stage of the pandemic coincided with the Spring Festival, and passengers mostly chose economical airline travel methods to save time and improve efficiency, so they were less sensitive to air fares, although the passenger load factor and load factor of economy airlines also declined compared with previous years. But compared with full-service airlines, economic aviation operations have not been significantly affected. With the gradual improvement of the domestic pandemic prevention and control situation, the demand for economical aviation has gradually recovered from the second quarter. Due to the increasingly severe international pandemic situation, the price of full-service airlines has increased significantly, making people's travel choices shift to economical aviation. Economy airlines has undergone a sudden change in the second half of 2020 and early 2021, but the increase in passenger demand makes the economy more economical. The passenger load factor of airlines have gradually recovered from the pandemic and maintained at a relatively high level, which has also promoted the increase of their ticket prices, making the coefficient rise instead of falling.

The estimation results are shown in the Table 5. The test results show that there is no change in the sign and significance level of the regression coefficient, which ensures the robustness of the change point identification and measurement estimation results.

**Table 5:** Robustness test results.

**Industry type**	**Company name**	**Parameter**	**Coefficient**	**Std. Dev**.	***t* statistic**	***P*-value**
Full server (Service type)	China Southern Airlines	α_*i*_	0.969	0.011	88.200	0.000
		β_*i*_	−0.028	0.016	−1.760	0.078
		γ_*i*_	0.207	0.076	2.740	0.006
	Eastern Airlines	α_*i*_	0.972	0.011	92.560	0.000
		β_*i*_	−0.015	0.011	−1.340	0.181
		γ_*i*_	0.147	0.057	2.600	0.010
	Air China	α_*i*_	0.789	0.019	41.690	0.000
		β_*i*_	−0.206	0.040	−5.190	0.000
		γ_*i*_	1.803	0.165	10.950	0.000
Economy (Low-cost type)	Spring and Autumn	α_*i*_	0.972	0.009	102.910	0.000
		β_*i*_	0.507	0.181	2.800	0.005
		γ_*i*_	1.156	0.387	2.990	0.003
	Huaxia	α_*i*_	0.981	0.009	109.130	0.000
		β_*i*_	0.092	0.057	1.620	0.106
		γ_*i*_	0.173	0.075	2.300	0.022
	Auspicious	α_*i*_	0.981	0.008	116.850	0.000
		β_*i*_	0.089	0.046	1.950	0.052
		γ_*i*_	0.215	0.097	2.210	0.027

## Discussion

### Main Findings

The main findings show that the Quandt-Andrews test of China's six full-service and economy airlines passed the significance test at a significance level of 1%. Secondly, there are industry differences between different airlines in the order of changes. The structural change point of full-service airlines affected by the pandemic is much earlier than that of economy airlines. In contrast, economy (low-cost) airlines have a certain lag in their change date, which is one point later than that of full-service airlines. More than quarterly, and the sudden change period of different companies in the economic type aviation industry is also quite different. The impact of the COVID-19 pandemic on China's full-service airlines is consistent, but the impact on economy (low-cost) airlines is different.

The impact of the pandemic on the stock prices of different types of airlines is also different. Structural changes caused by the impact of the pandemic have a significant impact on the stock prices of most airlines. The impact coefficients of the pandemic on full-service airlines are all negative. Among them, the pandemic has the largest negative impact on Air China, followed by China Southern Airlines and China Eastern Airlines the smallest. The impact of the COVID-19 pandemic on the three companies of Spring and Autumn, China Xia Airlines and Juneyao Airlines are all positive, and Spring Airlines has the greatest impact.

### Policy Implications

Under the external emergencies of the COVID-19 pandemic, economic aviation service companies and airlines that have taken active measures in response to major public health incidents have suffered much less negative impact than full-service airlines. Airlines should learn from them, reduce costs, increase efficiency, and optimize their endogenous mechanisms for responding to major public health incidents. The sustainable and healthy development of China's civil aviation industry ultimately depends on the endogenous development momentum established by the industry itself, rather than relying on external policies. Support should be good at transforming external forces into internal forces that promote the development of the civil aviation industry, and enhance the competitiveness of the civil aviation industry.

## Conclusion

This study assessed the analysis of the impact of the COVID-19 pandemic on the aviation industry as an opportunity, starting from a new perspective of airline service models, using daily data from full-service and economy airlines to study the impact of the COVID-19 pandemic on different types of airlines. Compared with the existing studies, in terms of the use of methods, for the research on the structural change of the stock price of the aviation industry in public emergencies, this paper relaxes the assumptions under the determined time point, and uses the Quandt-Andrews test to identify the change point, which provides a more effective tool for accurately evaluating and preventing the economic risk of the aviation industry. This paper first analyzes the impact of COVID-19's low cost and economy airline stock price by a new perspective of heterogeneous airline service model. It has made an empirical analysis on China Airlines for the first time. Other countries can use a larger sample size for further research on the same topic.

Although this article focuses on the impact of COVID-19 on the stock of different types of airlines in China, it has not been extended to more countries. In further research, the research period can be expanded and more companies can be added, and comparisons can be made from different countries to provide a more comprehensive economic impact of COVID-19 on the airlines industry.

## Data Availability Statement

The original contributions presented in the study are included in the article/supplementary material, further inquiries can be directed to the corresponding author.

## Author Contributions

YZ and LZ: methodology, data-processing, formal analysis, and writing-original draft preparation. FH: conceptualization, funding acquisition, and project management. All authors contributed to the article and approved the submitted version.

## Funding

This work was supported by National Bureau of Statistics of China (2021LY092).

## Conflict of Interest

The authors declare that the research was conducted in the absence of any commercial or financial relationships that could be construed as a potential conflict of interest.

## Publisher's Note

All claims expressed in this article are solely those of the authors and do not necessarily represent those of their affiliated organizations, or those of the publisher, the editors and the reviewers. Any product that may be evaluated in this article, or claim that may be made by its manufacturer, is not guaranteed or endorsed by the publisher.
